# P-2114. Prepared or Unprotected? Evaluating Live Viral Vaccine Coverage and Serologic Immunity Before Pediatric Solid Organ Transplantation

**DOI:** 10.1093/ofid/ofaf695.2278

**Published:** 2026-01-11

**Authors:** Benhur S Cetin, Caitlin N Brammer, William R Otto, Hilary Miller-Handley, Mark Murphy, Grant C Paulsen, Kerrigan Perkins, Teresa Ambrosino, Emily R Cain, Kathleen Campbell, Lara A Danziger-Isakov

**Affiliations:** Erciyes University, Kayseri, Kayseri, Turkey; Cincinnati Children's Hospital Medical Center, Cincinnati, OH; Cincinnati Children's Hospital Medical Center, Cincinnati, OH; Cincinnati Children's Hospital Medical Center, Cincinnati, OH; Cincinnati Children's Hospital Medical Center, Cincinnati, OH; Cincinnati Children's Hospital Medical Center, Cincinnati, OH; Cincinnati Children's Hospital Medical Center, Cincinnati, OH; Cincinnati Children's Hospital Medical Center, Cincinnati, OH; Cincinnati Children's Hospital Medical Center, Cincinnati, OH; University of Cincinnati College of Medicine, Cincinnati Children's Hospital Medical Center Division of Gastroenterology, Hepatology & Nutrition, Cincinnati, Ohio; Cincinnati Children's Hospital, Cincinnati, OH

## Abstract

**Background:**

Pediatric solid organ transplant (SOT) recipients are at increased risk for complications from vaccine-preventable diseases such as measles and chickenpox. Many recipients remain incompletely protected against these diseases when transplanted. Considering the recent measles outbreak, gaps in protection for live viral vaccines (LVVs) are increasingly important. This study assessed pre-transplant vaccination status for LVVs and immunity in SOT recipients.

**Table 1.**
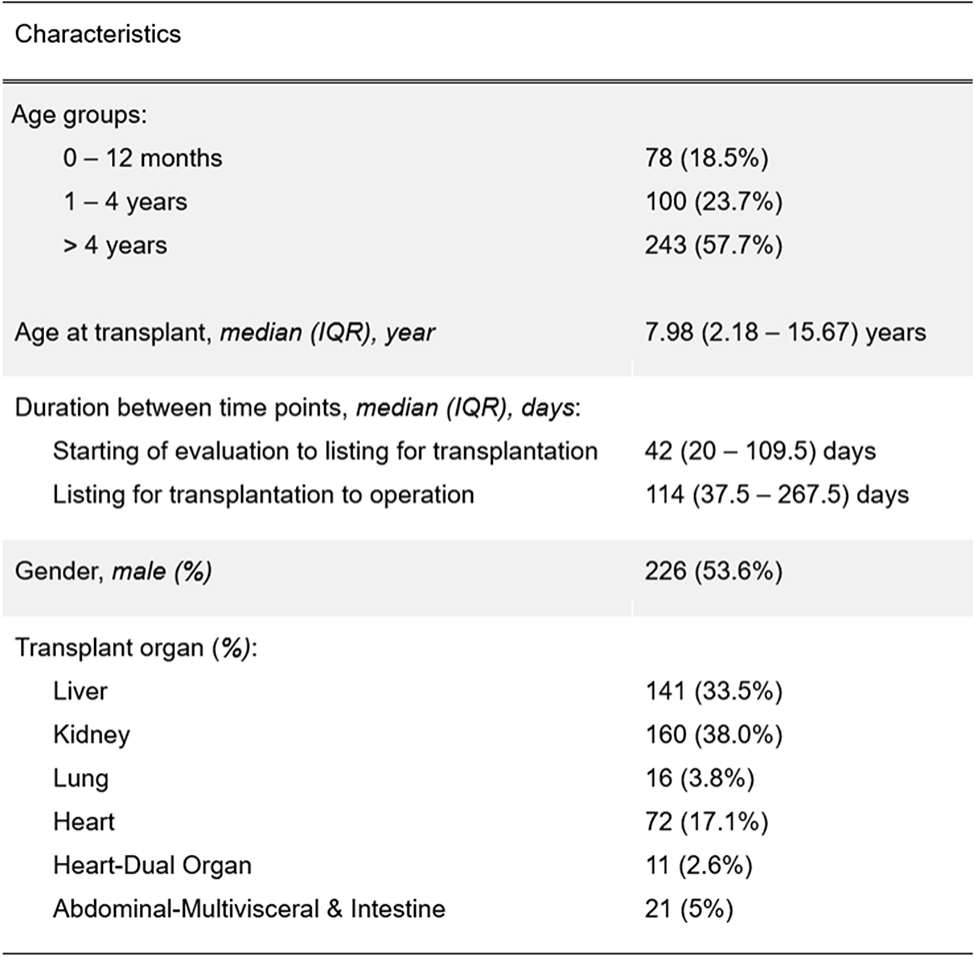
Demographics of pediatric solid organ transplant recipients.

**Table 2.**
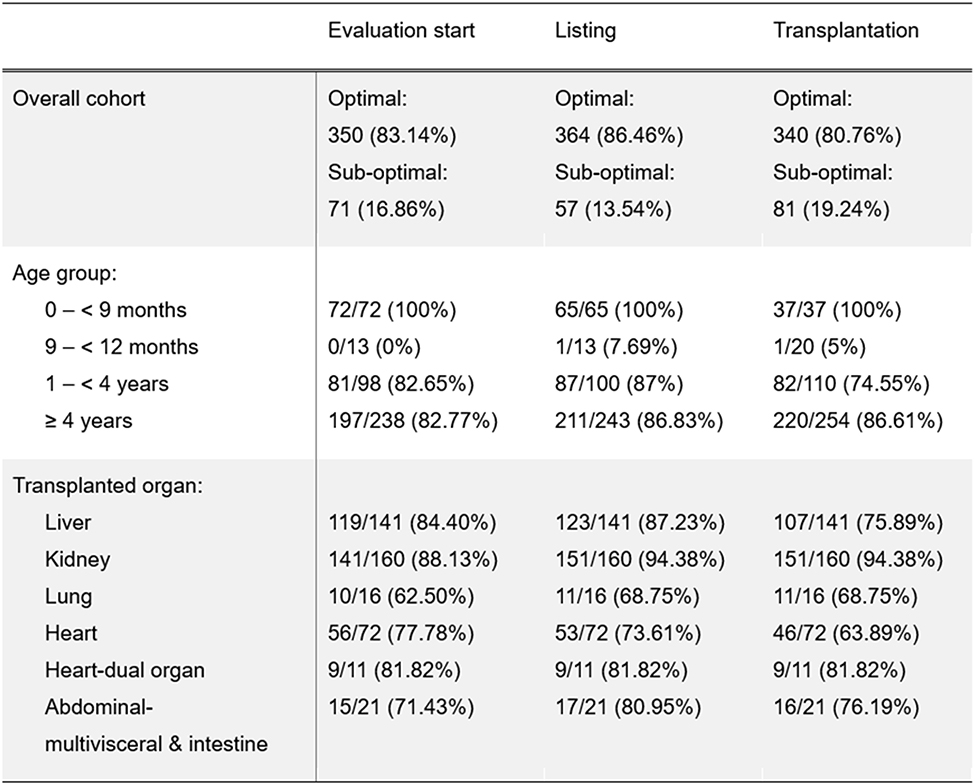
Varicella vaccination status of the recipients at different timepoints.

**Methods:**

We conducted a retrospective study of SOT recipients transplanted at Cincinnati Children’s Hospital Medical Center between 1.1.2018 and 12.31.2024. Demographics, vaccination history, and pre-transplant serologic results for varicella zoster virus (VZV) and rubeola were collected. VZV serology (IgG) was routinely collected pre-transplant with rubeola IgG added in early 2022. Patients were categorized by vaccination status and serological immunity at key pre-transplant timepoints.

**Table 3.**
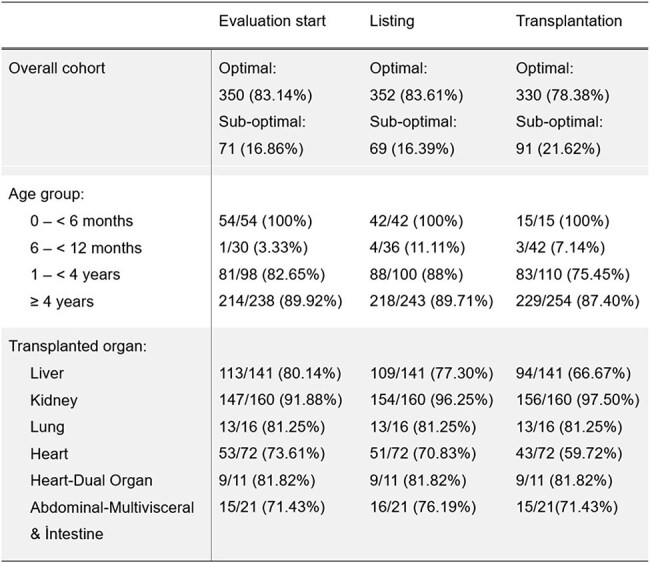
Rubeola vaccination status of the recipients at different timepoints.

**Results:**

A total of 421 patients were evaluated with liver and kidney most common (Table 1). The median (IQR) time between initiating transplant evaluation and listing was 42 (20 – 109.5) days, and the median time from listing to transplantation was 114 (37.5 – 267.5) days.

The rate of optimally vaccinated patients at evaluation, listing, and transplantation time was 83.1%, 86.5%, and 80.8% for VZV and 83.1%, 83.6%, and 78.4% for rubeola. The proportion of optimally vaccinated patients for VZV were lowest in lung (11/16) and heart (46/72) recipients, while for the rubeola rates were lowest in heart (43/72) and liver (94/141) recipients (Tables 2 & 3).

Serologic evaluation was performed in 94% of recipients for VZV and 58% of recipients for rubeola. Although serological responses were not checked after each vaccination, the pre-transplant seropositivity rates in optimally vaccinated patients were 76% (217/284) for VZV and 87% (168/193) for rubeola.

**Conclusion:**

In this study of pediatric SOT recipients, many patients are sub-optimally vaccinated with live viral vaccines prior to transplant. Optimal vaccination did not guarantee seropositivity. Our findings highlight the importance of early assessment, systematic vaccine documentation, and timely catch-up vaccination to improve protection against measles and varicella.

**Disclosures:**

Grant C. Paulsen, MD, Moderna, Inc: Grant/Research Support|Pfizer: Grant/Research Support|Sanofi: Grant/Research Support Lara A. Danziger-Isakov, MD, MPH, Aicuris: Grant/Research Support|Ansun BioPharma: Grant/Research Support|Astellas: Advisor/Consultant|Astellas: Grant/Research Support|Merck: Advisor/Consultant|Merck: Grant/Research Support|Pfizer (Any division): Grant/Research Support|Takeda: Grant/Research Support

